# A primer to vascular anatomy of the brain: an overview on anterior compartment

**DOI:** 10.1007/s00276-024-03359-0

**Published:** 2024-04-17

**Authors:** Diego Morales-Roccuzzo, Mohammadmahdi Sabahi, Michal Obrzut, Edinson Najera, David Monterroso-Cohen, Shadi Bsat, Badih Adada, Hamid Borghei-Razavi

**Affiliations:** Department of Neurological Surgery, Pauline Braathen Neurological Center, Cleveland Clinic Florida, 2950 Cleveland Clinic Blvd, Weston, FL 33331 USA

**Keywords:** Anterior cerebral artery, Internal carotid artery, Middle cerebral artery, Neurovascular anatomy, Vascular compendium

## Abstract

**Purpose:**

Knowledge of neurovascular anatomy is vital for neurosurgeons, neurologists, neuro-radiologists and anatomy students, amongst others, to fully comprehend the brain’s anatomy with utmost depth. This paper aims to enhance the foundational knowledge of novice physicians in this area.

**Method:**

A comprehensive literature review was carried out by searching the PubMed and Google Scholar databases using primary keywords related to brain vasculature, without date restrictions. The identified literature was meticulously examined and scrutinized. In the process of screening pertinent papers, further articles and book chapters were obtained through analysis and additional assessing of the reference lists. Additionally, four formalin-fixed, color latex-injected cadaveric specimens preserved in 70% ethanol solution were dissected under surgical microscope (Leica Microsystems Inc, 1700 Leider Ln, Buffalo Grove, IL 60089 USA). Using microneurosurgical as well as standard instruments, and a high-speed surgical drill (Stryker Instruments 1941 Stryker Way Portage, MI 49002 USA). Ulterior anatomical dissection was documented in microscopic images.

**Results:**

Encephalic circulation functions as a complex network of intertwined vessels. The Internal Carotid Arteries (ICAs) and the Vertebral Arteries (VAs), form the anterior and posterior arterial circulations, respectively. This work provides a detailed exploration of the neurovascular anatomy of the anterior circulation and its key structures, such as the Anterior Cerebral Artery (ACA) and the Middle Cerebral Artery (MCA). Embryology is also briefly covered, offering insights into the early development of the vascular structures of the central nervous system. Cerebral venous system was detailed, highlighting the major veins and tributaries involved in the drainage of blood from the intracranial compartment, with a focus on the role of the Internal Jugular Veins (IJVs) as the primary, although not exclusive, deoxygenated blood outflow pathway.

**Conclusion:**

This work serves as initial guide, providing essential knowledge on neurovascular anatomy, hoping to reduce the initial impact when tackling the subject, albeit the intricate vasculature of the brain will necessitate further efforts to be conquered, that being crucial for neurosurgical and neurology related practice and clinical decision-making.

## Introduction

It is paramount to understand the basis of neurovascular anatomy and this work aims to aid in the task of studying the brain and its nourishment from the ground up; given that, although the brain comprises less than 3% of the body’s weight, it receives around 15% of the cardiac output [41]. Average cerebral blood flow (CBF) is approximately 55 ml/100 grs of brain tissue/minute; being slightly more than 700 mL/min for a 1350 grs brain [41]. Therefore, neuronal cells have a high metabolic rate. Deprivation, even temporary, from oxygen and glucose might result in permanent brain damage and cell death.

Encephalic circulation can be thought of as a double inlet-single outlet vascular system: with two parallel arterial tandems, the pair of Internal Carotid Arteries (ICAs) and the pair of Vertebral Arteries (VAs) that form the Vertebrobasilar System (one providing the anterior circulation and the latter, the posterior circulation); and a main single outlet, the pair of Internal Jugular Veins (IJVs).

This complex layout is engineered so that each group of arteries is responsible for the irrigation of the wide and structure brimming Supratentorial Space and the more densely arrayed Posterior Fossa; the ICA and the VA, respectively. In other words, this so-called double inlet vascular system is comprised of the anterior circulation and posterior circulation, both of which are a crucial component of the Arterial Circle of Willis. Each circuit has its own territory, topography, singularities, and anatomical nuances. The foresaid circle of Willis lies in the basal aspect of the telencephalon and is responsible for supratentorial irrigation, owing its name to Thomas Willis, British neurologist and anatomist [12, 17, 44].

**Fig. 1:**
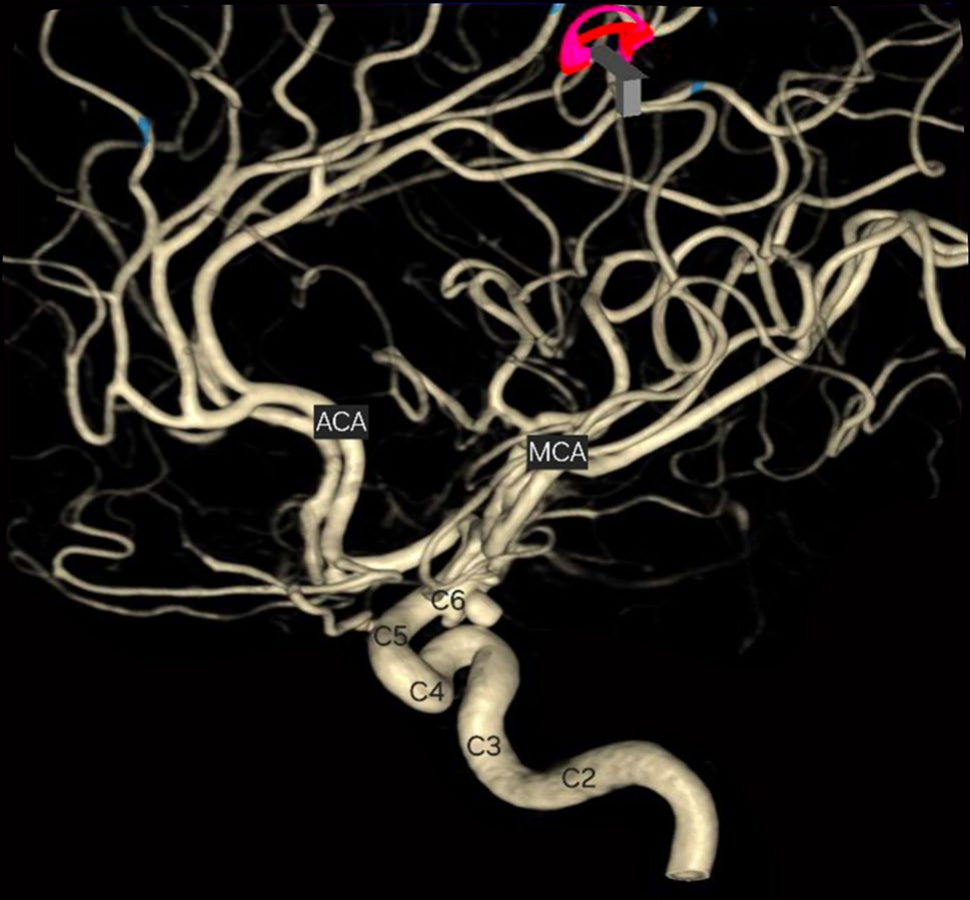
3D reconstruction of a cerebral angiogram from left Internal Carotid Artery, which depicts its segmentation (Bouthillier’s classification) and unveils an incidental aneurysm of C6 segment. *ACA* anterior cerebral artery, *MCA* middle cerebral artery

Comprehensive understanding of the vascular structure within the brain is crucial for effectively and safely treating various vascular conditions that impact the central nervous system. Among these conditions, aneurysms, arteriovenous malformations, and cranial dural arteriovenous fistulas have become a significant focus for both neurosurgeons and interventional neuroradiologists, employing a multidisciplinary approach [37]. The majority of these pathologies primarily affect the supratentorial region of the brain. Therefore, a profound knowledge of the microsurgical anatomy of the anterior circulation is essential for accurately locating the lesions using ancillary studies, as well as for laying out surgical strategies [8, 11, 19, 38, 39, 50] (Fig. 1).

Additionally, during procedures, identifying the different segments and branches of the (ICA), anterior cerebral artery (ACA), middle cerebral artery (MCA), and anterior communicating artery (ACommA) relies on recognizing specific landmarks in different topographic regions. This study attempts to provide a comprehensive review of the relevant anatomy of the anterior cerebral circulation in the context of managing neurovascular pathologies affecting the intracranial supratentorial region.

The lines that follow provide a robust framework for the neuroanatomy apprentice regarding the vasculature of the encephalon. It also aims to enhance the knowledge a general physician might have on the subject.

## Embryology

### Arterial embryology

It is fundamental to understand the embryology of the encephalic vascular system before one starts its study. Comprehending the embryological origins of the vessels can aid in understanding their anatomical variations and potential pathologies.

Around the 4^th^ week of gestation, the Central Nervous System starts developing its vascular structures, with the establishment of the neural tube. Numerous longitudinal low caliber vessels supply the neural tube via simple diffusion. The development of the aortic arch and great vessels takes place in two phases: the brachial and post-brachial phases [26, 41].

Whilst the first and second aortic arches regress, the third arch, the so-called carotid arch, gives rise to the internal carotid artery (ICA). The fourth aortic arch becomes the aortic arch per se, which persists into adult life, and then forms the right subclavian artery.

The ICA splits into the cranial division that becomes the ACA and the caudal division that becomes a precursor for the posterior communicating artery (PCommA). Through ependymal cells which invaginate into the central canal, the choroid plexuses are formed. Their stroma is derived from mesenchymal cells and the epithelial cells are born from the neuroepithelium. The anterior choroidal artery arises from the cranial division of the ICA and provides blood flow to the telencephalon. The caudal division gives rise to the superior cerebellar artery and gives supply to the cerebellum. In the hindbrain, the vertebrobasilar system is formed by the conjugation of the longitudinal neurovascular system. It supplies the developing brain, spinal cord, vertebral bodies, and duramater and gives rise to the future vertebral artery (VA) [20]. Moreover, there are carotid-vertebrobasilar anastomoses which temporarily provide arterial supply from the ICA to the longitudinal neural artery, the future vertebrobasilar artery in the hindbrain. The four known types are: the trigeminal, otic, hypoglossal and proatlantal intersegmental arteries. The arteries are accompanied by their corresponding nerves and resemble an intersegmental pattern. These vessels exist in the very early period of cerebral arterial development and rapidly involute within a week. Occasionally, persistence of the carotid to vertebrobasilar anastomosis is discovered in the adult period, and is considered a vestige of the corresponding primitive embryonic vessel [30].

### Venous embryology

The veins can be subdivided into the supratentorial, basal and an infratentorial venous systems [7, 14, 20, 32, 33, 45, 46].

The supratentorial venous system, on its own, is divided further into superficial and deep venous systems. Superficial and deep Sylvian veins are composed of embryological superficial and deep telencephalic veins that drain to the primitive tentorial sinus initially. The superficial Sylvian vein is hemodynamically balanced with the anastomotic vein of Trolard superiorly and the anastomotic vein of Labbè inferiorly [27]. The deep venous system initially forms as a drainage pathway of the choroid plexus system, which nourishes the early neural tube. Its drainage is through the median vein of the prosencephalon at 6–11 weeks of gestation. After regression of this vein, the adult deep venous system is established. Parenchymal venous drainages are either to the superficial veins through the superficial medullary veins or to the deep venous system (subependymal vein) through the deep medullary veins.

The basal system develops embryologically after the occlusion of the primitive tentorial sinus posteriorly, giving birth to the basal vein of Rosenthal, which receives not only the deep venous drainage, but also the superficial venous drainage. Therefore, it can be regarded as the superficial vein of the skull base, which receives deep venous drainage as well.

The infratentorial system is mainly comprised of three routes draining the brainstem and cerebellum. Anterior drainage (superior petrosal drainage) drains the archicerebellum, which is the basic drainage of the cerebellum through the embryological ventral metencephalic vein (trigeminal vein). Superior drainage (Galenic drainage) drains the paleocerebellum. Posterior drainage (torcular drainage) drains the neocerebellum. There are numerous transverse (transverse pontine veins) and longitudinal (anterior pontomesencephalic veins, anterior medullary veins, anastomotic lateral mesencephalic vein) anastomoses superficially between the three drainage routes. Like the cerebrum, the cerebellum has superficial and deep venous systems [20].

## Internal carotid artery

The ICA has been the subject of a multitude of studies and has been one of the most relevant and crucial vessels in the field of neuroanatomy, neurosurgery and neuroradiology. Several classifications exist that divide ICA in a certain number of segments. Keller, Rhoton, Bouthillier, are amongst the most prominent scholars that analyzed its intrinsic anatomy, relation with surrounding structures, microsurgical anatomy, histology, and in this fashion contributed to shape the most accepted and used classifications, of which Rhoton’s is the more widely spread one [25, 43]. According to it, the ICA can be divided in four segments, from C1 to C4, that go along with blood flow from the heart, from proximal to distal. These segments are: the Cervical ICA (C1), the Petrous ICA (C2), the Cavernous ICA (C3) and the Supraclinoidal ICA (C4) [43]. Moreover, C4 is further subdivided into Ophthalmic, Communicating and Choroidal segments. A handful of variant arterial pathways can be found throughout its course towards the intracranial compartment (retrojugular and/or retropharyngeal) and abnormal curvature (kinks, loops, and/or coils) might be encountered along its progress. Several collateral branches exist, often various either embryological persistent or inconstant amongst them [2]. Bouthillier’s classification is also widely spread and divides the ICA into 7 segments, them being: C1, cervical; C2, petrous; C3, lacerum; C4 cavernous; C5, clinoid; C6, ophthalmic; and C7, communicating. [3] For practical purposes, the authors will make use of Rhoton’s classification.

### Cervical ICA–C1

The Cervical ICA (C1), is the first and longest segment. It commences at the bifurcation of the Common Carotid Artery’s (CCA). Usually at the levelof the fourth cervical vertebrae at Farabeuf’s triangle, which is delineated by the internal jugular vein (IJV) posteriorly, the thyrolinguofacial venous trunk anteriorly, and the hypoglossal nerve superiorly. It lies deep to the medial border of the sternocleidomastoid muscle, and it relates laterally with the internal jugular vein and posteriorly with the X cranial nerve, wrapped along the other structures in the carotid sheath. Along with it, postganglionic sympathetic fibers (PGSNs) ascend to form the Internal Carotid Plexus [4, 12].

It contains the carotid sinus, a crucial baroreceptor that senses changes in systemic blood pressure and is located in the adventitia of the carotid bulb of the internal carotid artery, which is intimately related but a distinct organ from the carotid body, a chemoreceptor found at the CCA’s bifurcation. It arises towards the carotid canal of the petrous bone at the exocranial skull base and on its way, the ICA passes through the parapharyngeal space which is divided into pre-and post-styloid compartments and bordered laterally by the posterior belly of the digastric muscle. Often overlooked, this segment holds utmost significance, particularly when addressing lesions such as Glomus Jugulare. The surgical implications can indeed be dreadful. Consequently, it can be localized along the exocranial paramedian surgical corridor (one of three skull-base surgical corridors postulated as modular compartments: median, paramedian and lateral), as meticulously described by impeccable works [10, 13]. 

### Petrous ICA–C2

The Petrous segment, still surrounded by PGSNs, begins when the ICA enters the carotid canal of the petrous bone and ends in the internal orifice of the carotid canal. Three subdivisions can be identified: (a) a shorter one (vertical) that ascends and then bends in a rostral direction; (b) a posterior loop, anterior to the cochlea; (c) and a horizontal longer segment (each segment connected by genu, the posterior and the anterior genu). The latter is intimately related with the V cranial nerve, lying inferomedial to Meckel’s cave [23], where the Gasserian ganglion can be found, in the proximity of the VI nerve’s entrance to Dorello’s canal (below the posterior clinoid process, superior to the petroclival venous confluence [8, 21, 22]); afterwards it traverses in an antero-medial direction, heading to the apex of the petrous bone, parallel to its sagittal axis, to finally reach the aforementioned internal carotid canal orifice, and enter the cavernous sinus anteriorly to petrosphenoidal ligament or “Grubert’s ligament”, which extends from the petrous apex to the petrosal process of the sphenoid bone. Therefore, the horizontal portion, also related to the greater and lesser superficial petrosal nerves, surpasses the foramen lacerum (comprised by the union between the petrous apex, the lateral aspect of the dorsum sellae, and the sphenoid body) forming the anterior genu and ascends with a parasellar trajectory, emerging from the internal carotid canal, piercing the dura as it passes the petrolingual ligament, which bonds the petrous apex and the lingual process of the sphenoid bone, to become the next carotid segment [5, 9, 43].

In the petrous ICA it has also been described a thick “carotid cuff” [44], formed by connective tissue around the artery and a venous plexus. The origin of two branches can be identified from this portion, the caroticotympanic artery (which is inconsistent) and the pterygoid branch (present in approximately 30%) [30]. Additionally, it is worth noting that roughly in more than 80% of cases, there is a dorsal (endocranial) dehiscence of the petrous carotid canal, which can have critical clinical implications [16, 34].

### Cavernous ICA–C3

Once the ICA emerges from the carotid canal and traverses below the petrolingual ligament, which extends from the petrous apex to the lingual apophysis of the sphenoid bone, it enters the cavernous sinus running through the carotid sulcus, hence becoming the intracavernous ICA. This segment, from proximal to distal, can be divided into four segments: (1) the short vertical segment (a continuation of the paraclival ICA); (2) the posterior genu; (3) the horizontal segment; and (4) the anterior genu, which continues with the paraclinoidal ICA as it emerges from the CS. The intracavernous ICA is covered by a vascular membrane lining the sinus, still surrounded by PGSNs [43]. This portion of the ICA is intimately related to the medial wall of the cavernous sinus, of utmost clinical importance: In the nearest vicinity lies the significant carotico-clinoid, inferior parasellar and posterior parasellar ligament.

The cavernous carotid segment is directed anteriorly and then supero-medially, to bend posteriorly forming in this manner the posterior loop of ICA. The ICA continues horizontally and afterwards bends towards a rostral direction (part of anterior loop of ICA) heading to the anterior clinoid process. Afterwards, it traverses the proximal dural ring—namely the “carotid-oculomotor membrane” (which incompletely encircles the ICA), given the strict adjacency between the ICA and the CN III or oculomotor nerve-, and pierces the dura at the distal dural ring (which completely encircles ICA) where the artery becomes intradural. The segment of the ICA that is found between the proximal and distal dural rings, these also defining the limits of the carotid collar, is additionally known as the clinoid segment. The clinoid segment is bounded medially by the carotid sulcus, anteriorly by the optic strut, and medially by the anterior clinoid process. The distal dural ring is tightly adherent to the anterior and lateral aspect of the clinoid segment, but not the medial and posterior aspect. The posteromedial intradural area around the distal clinoid segment is known as the carotid cave [4, 5, 12, 18, 43, 53].

The cavernous carotid artery has many branches, usually grouped as trunks. The main trunks and branches are the following: (A) Meningohypophyseal trunk, which the largest & most proximal, emerging at the level of the posterior loop and present in 100% of cases. Meningohypophyseal trunk has three branches: The posterolateral branch, tentorial artery (or artery of Bernasconi & Cassinari): provides the blood flow to the proximal III CN, proximal IV CN and Gasserian ganglion [28]. (This branch can often supply petroclival meningiomas.) The posteromedial branch, dorsal meningeal artery (or dorsal clival a.) supplies the proximal VI CN and, seldom, the III CN. Finally, a medial branch giving rise to the inferior hypophyseal artery which supplies the posterior lobe of the hypophysis. It is worth noting that this trunk’s branches can emerge directly from the Internal Carotid Artery. (B) Anterior meningeal artery (C) Inferolateral trunk (ILT), another constant collateral (present in > 90%), which provides supply to the cranial nerves that travel through the inner layer of the cavernous sinus’ lateral wall [6, 8, 18, 28, 47, 50, 53]. All of its branches terminate on the inferomedial aspects of the intracavernous CNs. Extensive anastomoses have been found between the ILT branches and the branches arising from external carotid artery. These anastomoses supply the III CN, proximal IV CN, distal VI CN and proximal V_1_ and V_2_ CN [28]. (D) At last, a medial branch is found in less than 10% of cases, the capsular artery of McConnell, which supplies the capsule of the pituitary gland [8, 15, 24, 29, 40, 47].

### Supraclinoidal ICA–C4

Once the ICA traverses the distal dural ring, it is known as Supraclinoidal ICA. It turns upwards, posteriorly and laterally, toward the lateral aspect of the optic chiasm, then up to the anterior perforated substance to end up bifurcating just below the anterior perforated substance into two terminal branches. One branch extends anteromedially, the anterior cerebral artery. The second branch extends laterally, forming the middle cerebral artery. According to Dr. Rhoton’s classification, C4 Supraclinoidal segment is subdivided into three divisions, namely the ophthalmic, the communicating and the choroidal division [4, 12, 43].

#### Ophthalmic segment

The longest portion of C4 commences immediately distal to the dural ring and ends just proximal to the origin of the Posterior Communicating Artery (p-comm). Important perforating arteries arise from this portion, supplying the optic nerve and other chiasm related structures. Two main collaterals are typically present, including the Ophthalmic Artery and Superior Hypophyseal Artery. In more than 96% of cases, the Ophthalmic Artery emerges from the ventral aspect of the Supraclinoidal ICA just inferior to the optic nerve and the anterior clinoid, distal to the cavernous sinus. In the remaining 4%, the ophthalmic artery arises from the cavernous segment. In general, the origin of the ophthalmic artery if often taken to mark the location of the carotid artery entrance into the subarachnoid space, and serves as an important landmark in angiography. The origin of the ophthalmic artery from the ICA can vary by 5 mm anterior to 7 mm posterior to the anterior clinoid process, of which it keeps intimate vicinity. The ophthalmic artery then runs through the optic canal into the orbit, superomedial to the optic strut, along with the II cranial nerve, inferolateral to the latter. This artery has a conspicuous “kink” that can be seen on lateral angiograms [31]. Regarding the Superior Hypophyseal Artery, in the medial wall of the ICA several of the aforementioned perforator arteries emerge, destined to the pituitary stalk and anterior lobe of the pituitary gland. One of the branches is usually and also supplies the dura of the cavernous sinus.

#### Communicating segment

The Communicating Segment begins immediately proximal to the Posterior Communicating Artery origin and traverses between II and III cranial nerves, to terminate at the origin of the Anterior Choroidal Artery. Its branches include the PCommA and the Anterior Thalamoperforator Arteries. The PCommA arises from the posterior-medial wall of the ICA, and from there it extends dorsal and slightly medial to encounter Posterior Cerebral Artery, lateral to the optic tract and the mamillary bodies, rostral and dorsal respectively.

The PCommA can also be found as a variant in nearly 18% of cases, known as Fetal p-comm, which has a greater diameter than the proximal P1 segment of the ipsilateral posterior cerebral artery thus providing the majority of blood flow to its respective territory. Several perforators emerge from the PCommA and can be divided into two groups: Anterior perforating group and Posterior perforating group, which are destined to the hypothalamus, ventral thalamus, posterior perforated substance and subthalamic nucleus. Anterior Thalamoperforator Arteries irrigate the optic tract, the optic chiasm, the posterior hypothalamus, the posterior limb of the internal capsule and the subthalamus. Also referred to as premamillary arteries, they often arise from the PCommA. A number of these structures are featured in anatomical dissections and figures all along the text of this article.

#### Choroidal segment

**Fig. 2:**
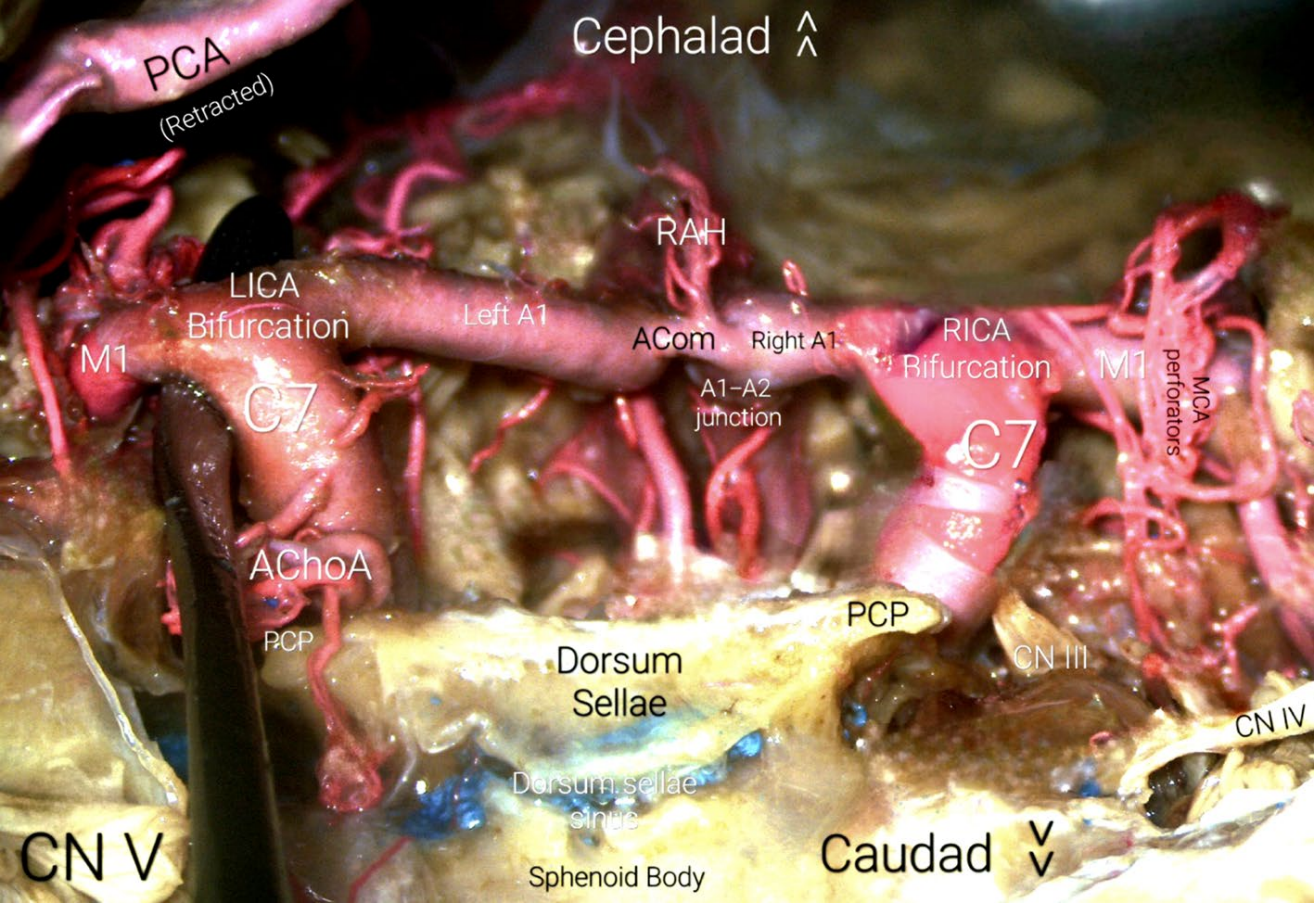
Posterior view of the Circle of Willis’ anterior half. *Acom* ACommA, anterior communicating artery, *AChoA* anterior choroidal artery, *LICA* left ICA, *MCA* middle cerebral artery, *PCA* posterior cerebral artery, *PCP* posterior clinoid process, *RAH* recurrent artery of Heubner, *RICA* right ICA

**Fig. 3:**
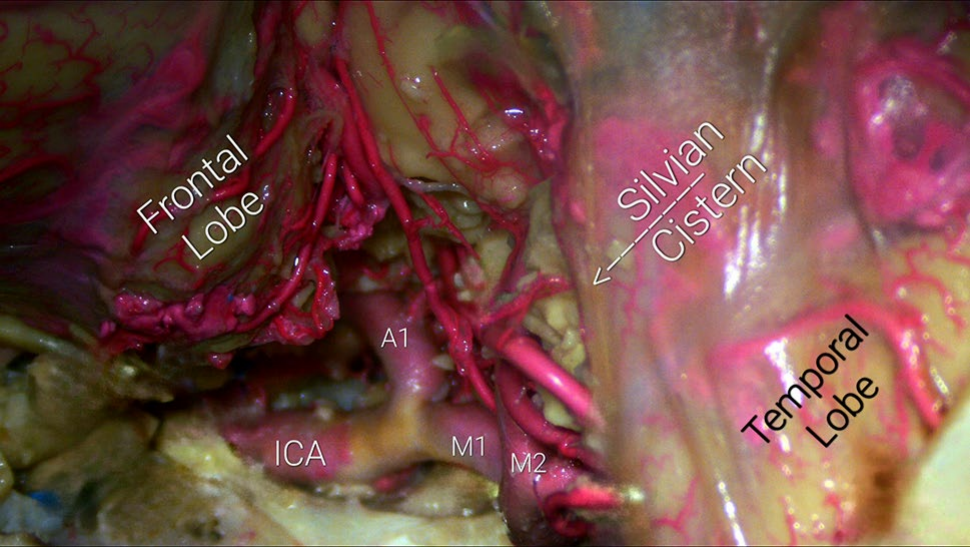
Terminal ICA bifurcation into A1 and M1, deep in the carotid cistern. It is shown the utilized direction of dissection of the Silvian Cistern's arachnoid

**Fig. 4:**
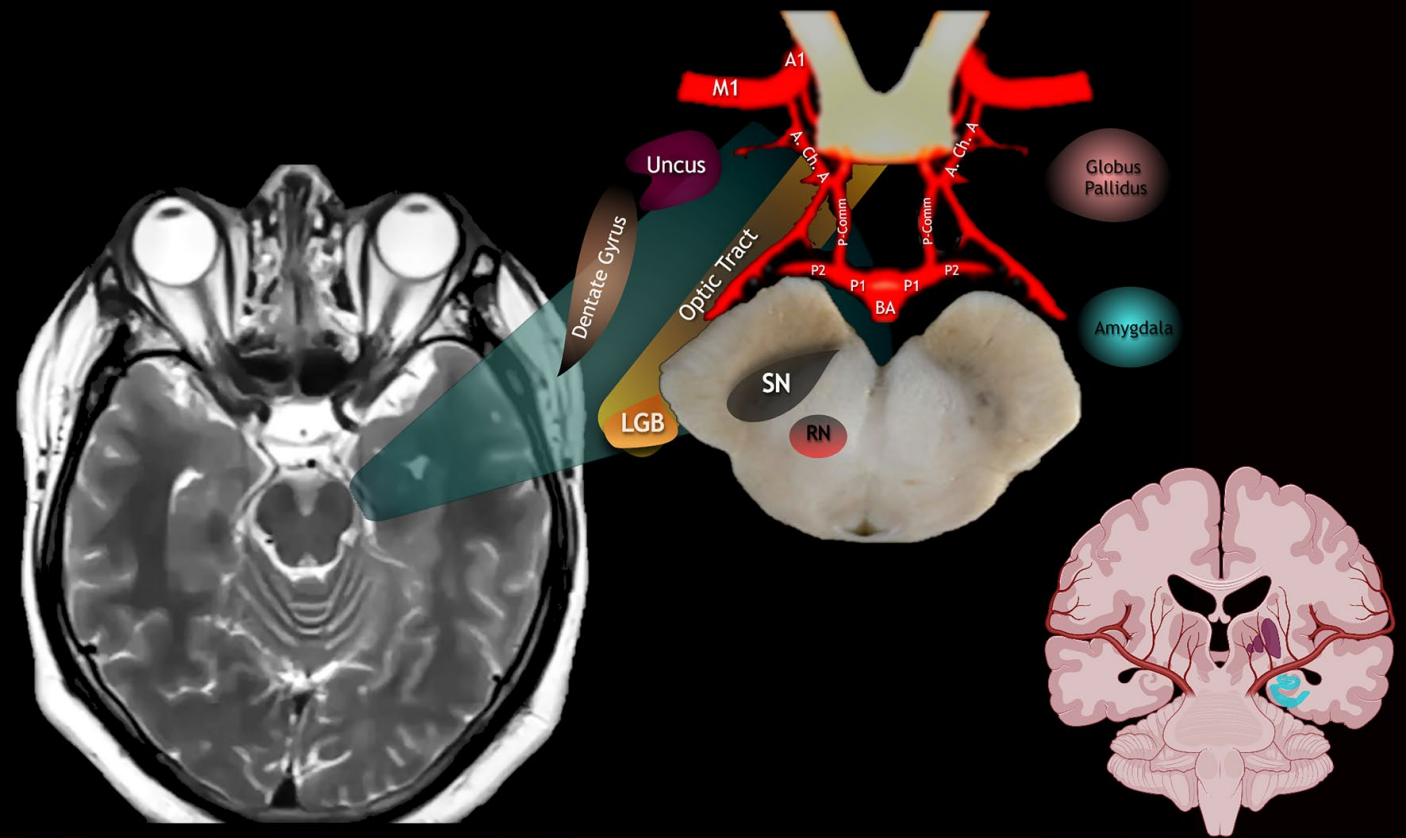
Schematically illustrated territories and deep brain structures supplied by the anterior choroidal artery and the main blood supply territories and structures nurtured by perforating arteries arising from A1 and M1. Recurrent Artery of Heubner not depicted, although crucial for midline supply of Central Core structures. *SN* substantia nigra, *RN* red nucleus, *LGB* lateral geniculate body

The Choroidal segment spans from the Anterior Choroidal Artery (A.Ch.A.) origin to the bifurcation of the ICA into the ACA and MCA. The Anterior Choroidal Artery originates from the posterior-lateral wall of the ICA. It takes off is usually 2 to 4 mm distal to PCommA and courses posteriorly toward the lateral aspect of the lateral geniculate body. It then directs itself laterally, inferior to the optic tract, to reach the inferior choroidal point, to pierce the temporal horn and anastomose which branches from the posterolateral choroidal artery. One can recognize two portions, the cisternal and plexal segments. The cisternal segment, extends from its origin to the inferior choroidal point and runs through the crural cistern, parallel to the optic tract within it. Limited anterolaterally by the uncus and posteromedially by the cerebral peduncle, it is further divided into proximal and distal cisternal A.Ch.A., according to the relation with the lateral geniculate body. The plexal segment enters the supracornual recess of temporal horn, penetrating the choroidal point. It mainly but not exclusively supplies crucial structures of the diencephalon, mesencephalon, central core and mesial temporal lobe, such as: portion of optic tract, medial globus pallidus, genu of the internal capsule, the inferior half of posterior limb of IC, uncus, retrolenticular fibers (optic radiation), lateral geniculate body, dentate gyrus, fornix, globus pallidus, red nucleus, locus niger, amygdala, temporal horn, lateral geniculate body, hippocampus, and anterior perforated substance. Figures 2, 3 and 4.

## Anterior cerebral artery (ACA)

The ACA is one of the two paired terminal branches of the ICA, emerging from its anteromedial wall and traversing anteriorly over the optic chiasm, below the anterior perforated substance. It then curves anteriorly to extend along the corpus callosum towards. At their most distal extent, the posterior branches of the ACA anastomose with the distal branches of the Posterior Cerebral Artery over the splenium of the corpus callosum. The ACA is one of the vessels that form the anterior half of the Circle of Willis and each one is connected to its contralateral homologue by the Anterior Communicating Artery (ACommA), that links them immediately above the optic chiasm and pituitary stalk. Thus, one can recognize two segments: the pre-communicating and post-communicating. The latter is further divided into several segments, which will be detailed later on. Is in this manner that after its origin, the ACA courses medially to pass above the chiasm or optic nerve and below the lateral olfactory stria to reach the basal aspect of the interhemispheric fissure where it joins the ACommA [35].

The ACA mainly provides blood flow to the anterior and medial (parafalcine) aspect of the brain hemispheres: superior frontal gyrus, the superior parts of the precentral, central and postcentral gyri; but it is also key to the irrigation of certain structures of the central core (part of globus pallidus, putamen, internal capsule anterior limb, caudate nucleus), the diencephalon, the corpus callosum, olfactory bulb and tract, optic chiasm and a fairly important portion of frontal lobe’s basal aspect, the medial part of the orbital gyri, gyrus rectus, paraolfactory gyrus and paraterminal gyrus (area furthermore known as subcallosal area).

**Fig. 5:**
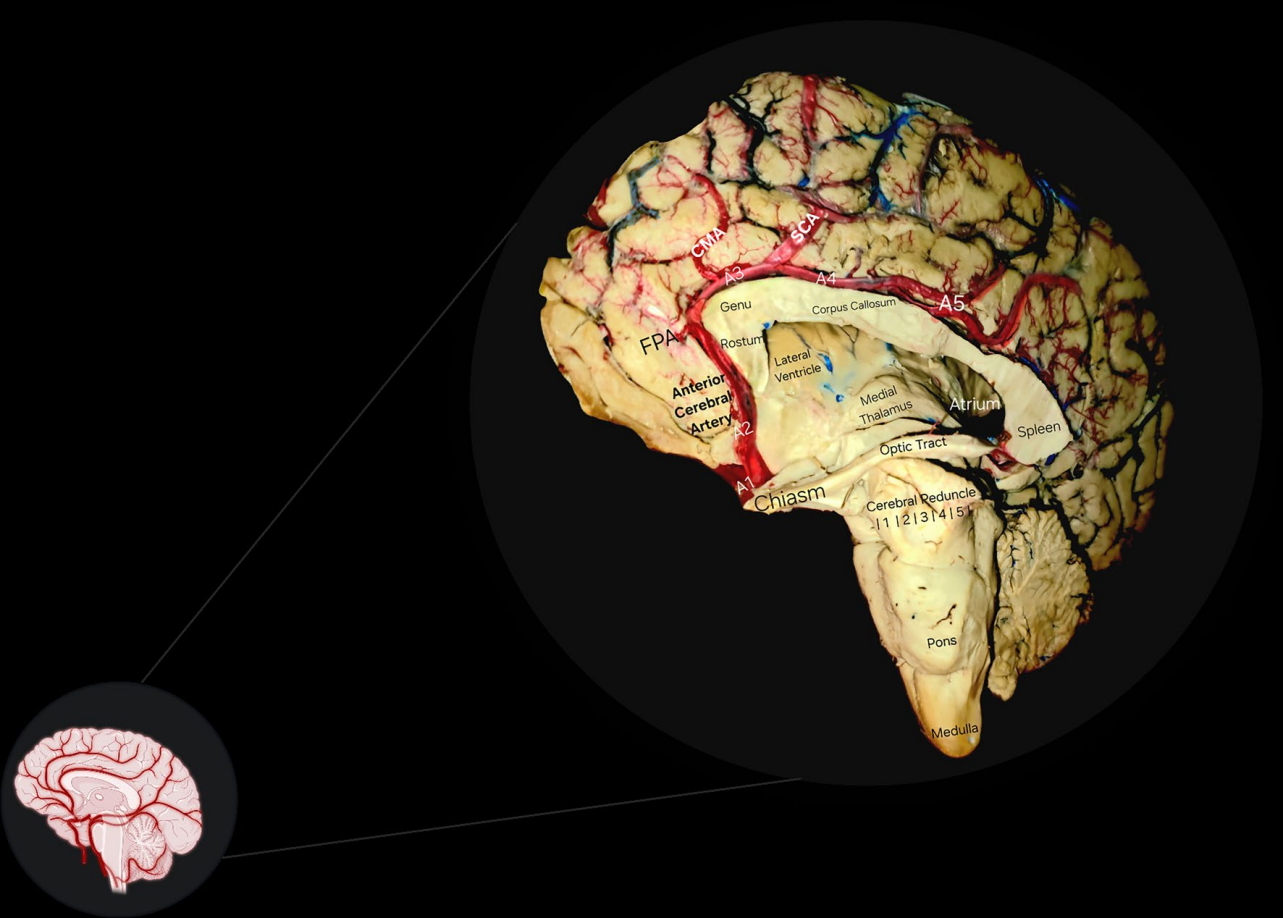
In situ anatomy of the Anterior Cerebral Artery with its segments. Worth noting, an ealy Supracallosal artery is observered supplying the cingulum, whilst the remaining main segments of the ACA continue their path above the corpus callosum. Some of the main neural structures have been carefully depicted for educational purposes, including the divisions of the cerebral peduncles (Cortico-spinal, Cortico-bulbar, Cortico-pontine, Parieto-occipito-temporo-pontine, and Fronto-pontine projections). *FPA* fronto-polar artery, *CMA* callosal-marginal artery, *SCA* supracallosal artery

It has little expression on the convexity, however it correlates directly to Penfield’s Homunculus sensory-motor functional areas facing the interhemispheric fissure, such as the lower extremities and the genitals. Its average diameter at the rising point is 2.5 mm [29] (Fig. 5).

The ACA can be segmented as follows:

### A1 (Pre-communicating)

From origin to Anterior Communicating Artery, typically with a length of 12–13 mm.

### A2 (Post-communicating)

From ACommA to the origin of the callosomarginal artery. The section that lies beneath the genu of the corpus callosum, is referred to as subgenual portion, which holds importance regarding angiographic diagnosis of aneurismatic pathology.

### A3 (Precallosal)

From the callosomarginal artery’s branch point, curving around the genu of the corpus callosum to its superior surface, less than 5 cm posterior to the genu.

### A4 (Supracallosal)

Leveled with the body of the CC until an imaginary line continued with the coronal suture.

### A5 Terminal branch (Postcallosal)

In the proximity of the splenium. It is also known as the posterior supracallosal, whereas the A4 segment is commonly referred to as the anterior supracallosal.

Altogether, the A2 to A5 segments are also referred to as the pericallosal artery. The pericallosal artery is located below the free margin of the falx in all its portions but the most posterior, this aspect being crucial for guiding oneself during surgical interventions of pericallosal artery aneurysms.

**Fig. 6:**
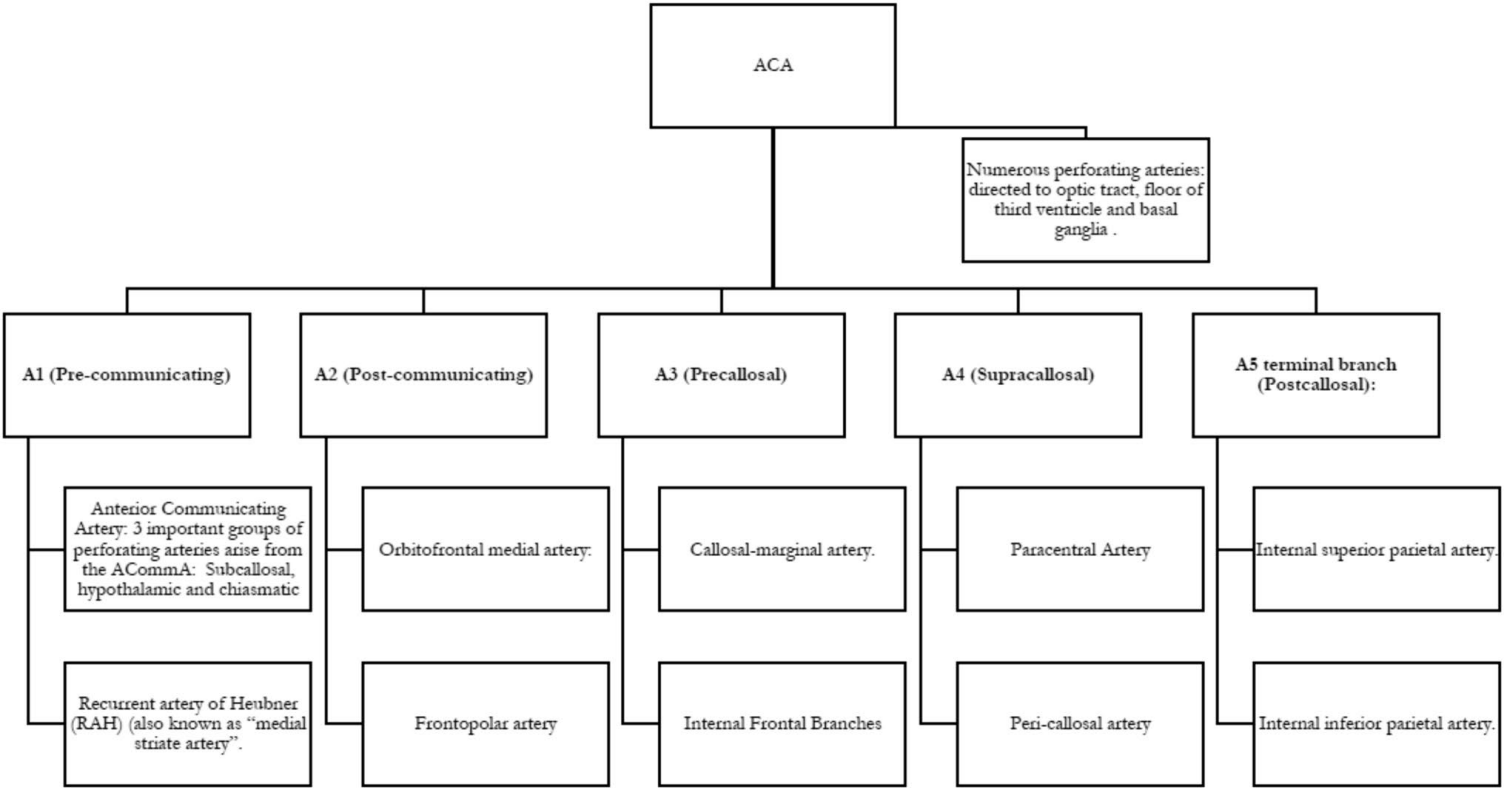
Anterior Cerebral Artery Segmentation

The constant branches usually found in the ACA are [29, 36, 43]: (a) Anterior Communicating Artery. Typically, average diameter and length: 1.5 mm and 4 mm, accordingly. Three important groups of perforating arteries arise from the ACommA: subcallosal, hypothalamic and chiasmatic. (b) Recurrent artery of Heubner (RAH) (also known as “medial striate artery”): The more conspicuous perforating vessel, it runs through the anterior third of the anterior perforated substance and heads to the basal ganglia and the internal capsule, only after it divides into several branches. It can be defined as “the ACA perforating branch arising from proximal A2, ACommA, or distal A1 segment that curves back sharply on itself, paralleling the A1 segment, and passing above the carotid bifurcation into the medial part of the Sylvian fissure before entering the anterior perforated substance”. (c) Orbitofrontal medial artery: is the ACA cortical branch that supplies the gyrus rectus, olfactory bulb-tract, and orbital gyri. (d) Frontopolar artery, cortical branch that supplies the ventromedial frontal lobe. (e) Callosal-marginal artery (Internal Frontal Branches and Paracentral Artery). (f) Peri-callosal artery (Internal superior parietal artery and Internal inferior parietal artery). (g) Perforating arteries: numerous perforating arteries arise from the pre- and post-communicating segments and run through the anterior perforated substance. These are directed to the optic tract, floor of the third ventricle and the basal ganglia (Fig. 6).

## Middle cerebral artery (MCA)

**Fig. 7:**
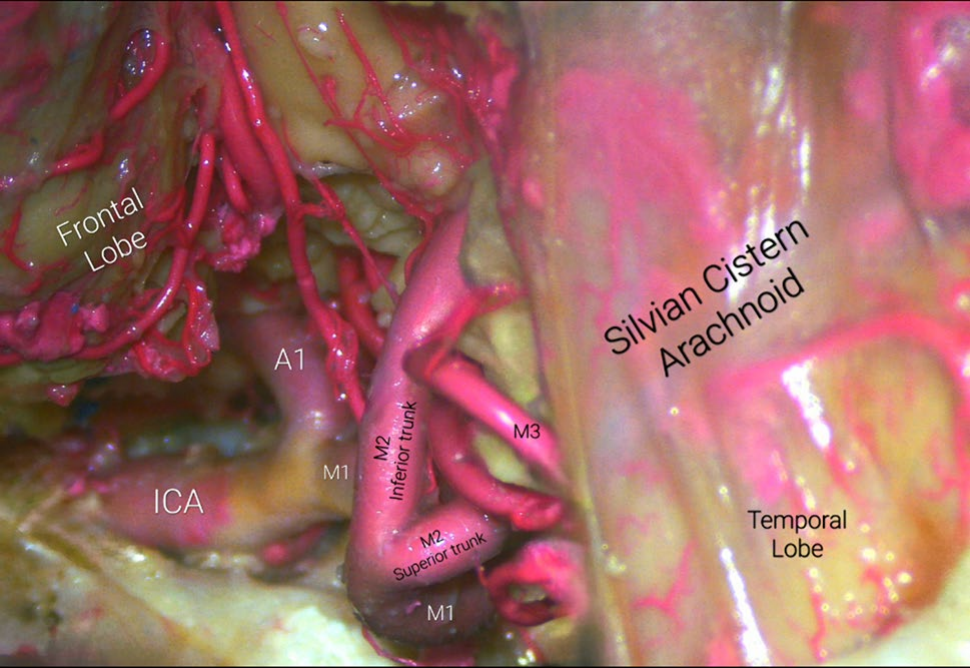
Bifurcation of Middle Cerebral Artery into superior and inferior trunks

The MCA is the lateral terminal branch of the ICA. In most cases it originates from the anterolateral wall of the ICA in the carotidal cistern below the anterior perforated substance, and heads laterally to the Silvian cistern [51], horizontally and parallel to the axial plane, behind the sphenoid ridge to reach the limen insula, turning aggressively in a cephalad direction. From that point it splits into two, three or four trunks and turns back and upward to traverse across the Silvian fissure and through the insular lobe surface [52]. As it navigates through the insular cortex bounded by the circular sulcus, it reaches frontal, temporal and parietal opercula; and gives several opercular branches that head in cephalad and caudate directions respectively to provide the convexity of the brain with blood flow. However, the MCA can also provide what are known as early branches: Uncal and temporopolar arteries and the anterior temporal artery, are examples of early cortical branches [4, 12, 43] (Fig.  7).

MCA is usually divided into 5 portions.

### M1 (Sphenoidal)

From its origin to the point where it bends towards the insular surface in a 90° angle at the level of the limen insulae. It bifurcates into inferior and superior trunks in approximately 80% of cases. A trifurcation can be found in 15% and a tetrafurcation or division into multiple lesser trunks, occurs in less than 5% of cases. The bifurcation further divides the M1 segment into pre-bifurcation and post-bifurcation segments, the latter of which has a superior and inferior trunk.

### M2 (Insular)

MCA trunks from bifurcation to emergence from Sylvian fissure. This segments traverses across the inferior circular sulcus of the insula.

### M3 (Opercular)

After several branches emerge and run dorsally across the sulci of the insula, another 90° turn is described as the MCA branches travel through the opercular surface of the temporal and fronto-parietal lobes.

### M4 (Cortical)

The most distal segment, it distributes itself all along the convexity of the brain, providing blood supply to the superolateral and temporal surfaces.

### M5 (Cortical terminal branches)

Occasionally, the most distal terminal branches are referred to as M5, as a common group.

**Fig. 8:**
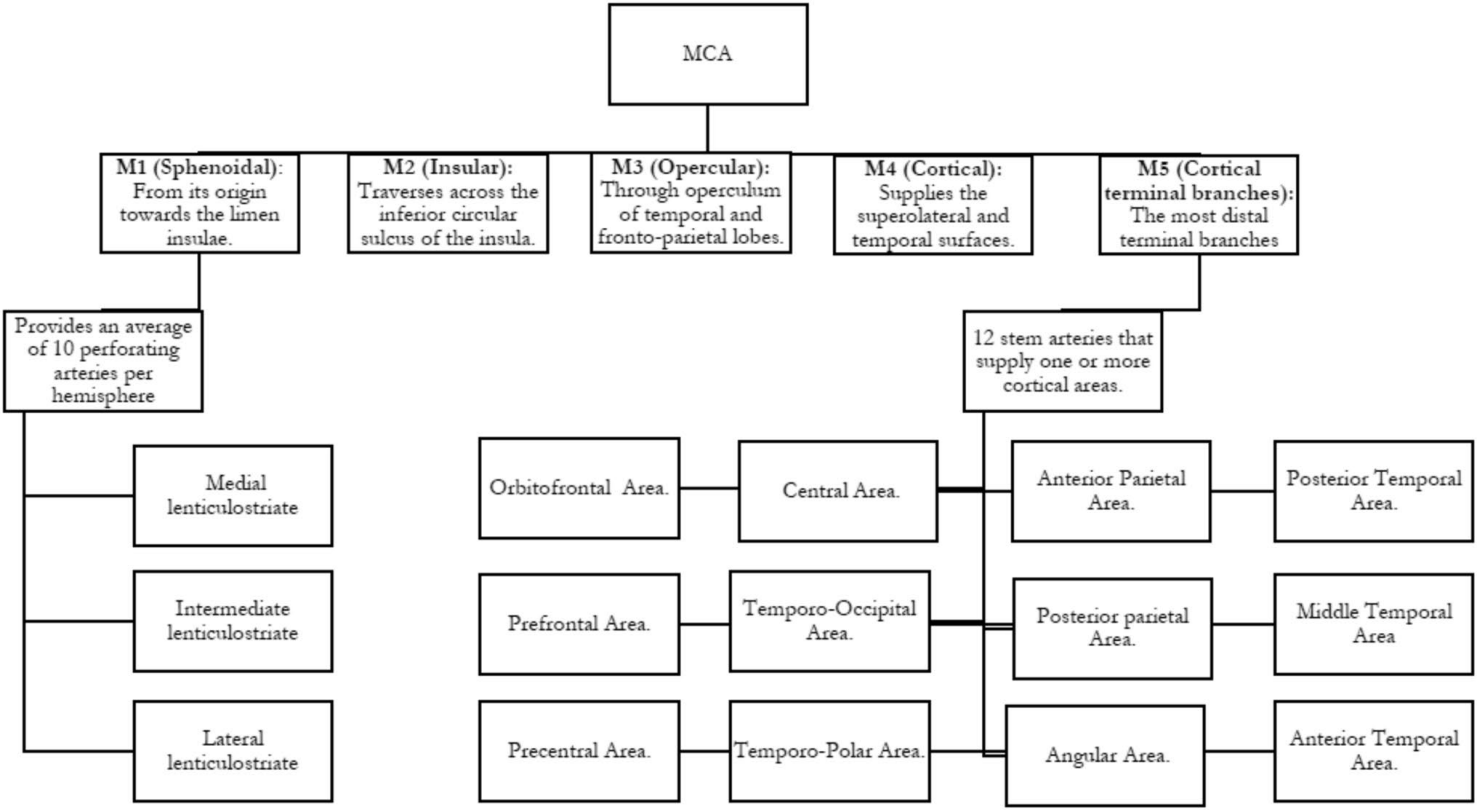
Middle Cerebral Artery Segmentation

**Fig. 9:**
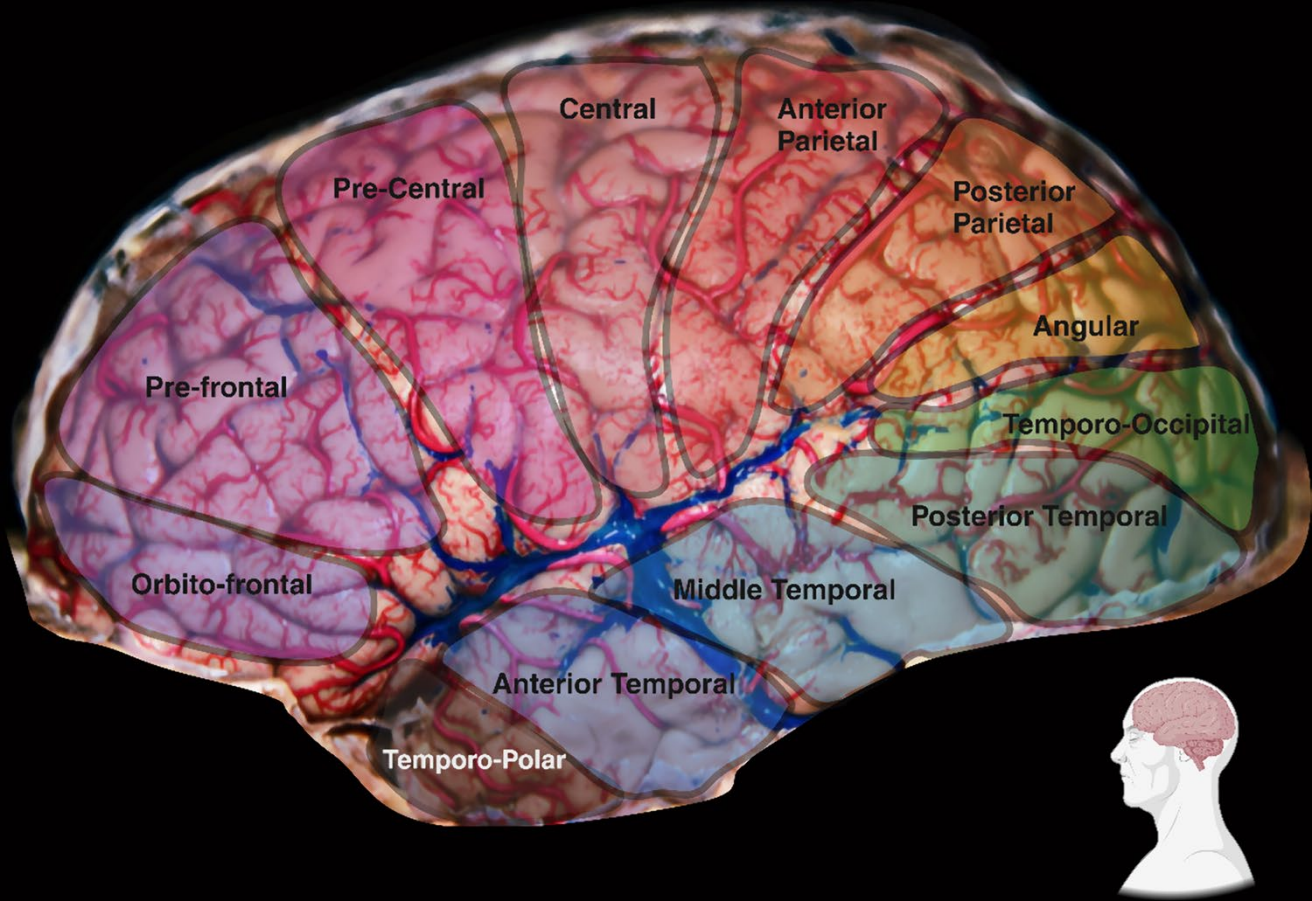
A view of cortical branches of Middle Cerebral Artery, especially opercular branches, and the Superficial Middle Cerebral Vein receiving drainage from Vein of Labbé. Shown is the segmentation according to stem artery territories, as discussed in the text

According to Rhoton’s investigation, there are 12 cortical areas irrigated by the MCA and each one of them is supplied by a stem artery that, in fact, supplies one or two more cortical areas. Only the temporo-occipital, angular and central areas have exclusive arterial stems. They can be listed as follows: Orbitofrontal Area. Prefrontal Area. Precentral Area. Central Area. Anterior Parietal Area. Posterior Area. Angular Area. Temporo-Occipital Area. Posterior Temporal Area. Middle Temporal Area. Anterior Temporal Area. Temporo-Polar Area (Figs. 8 and 9).

**Fig. 10:**
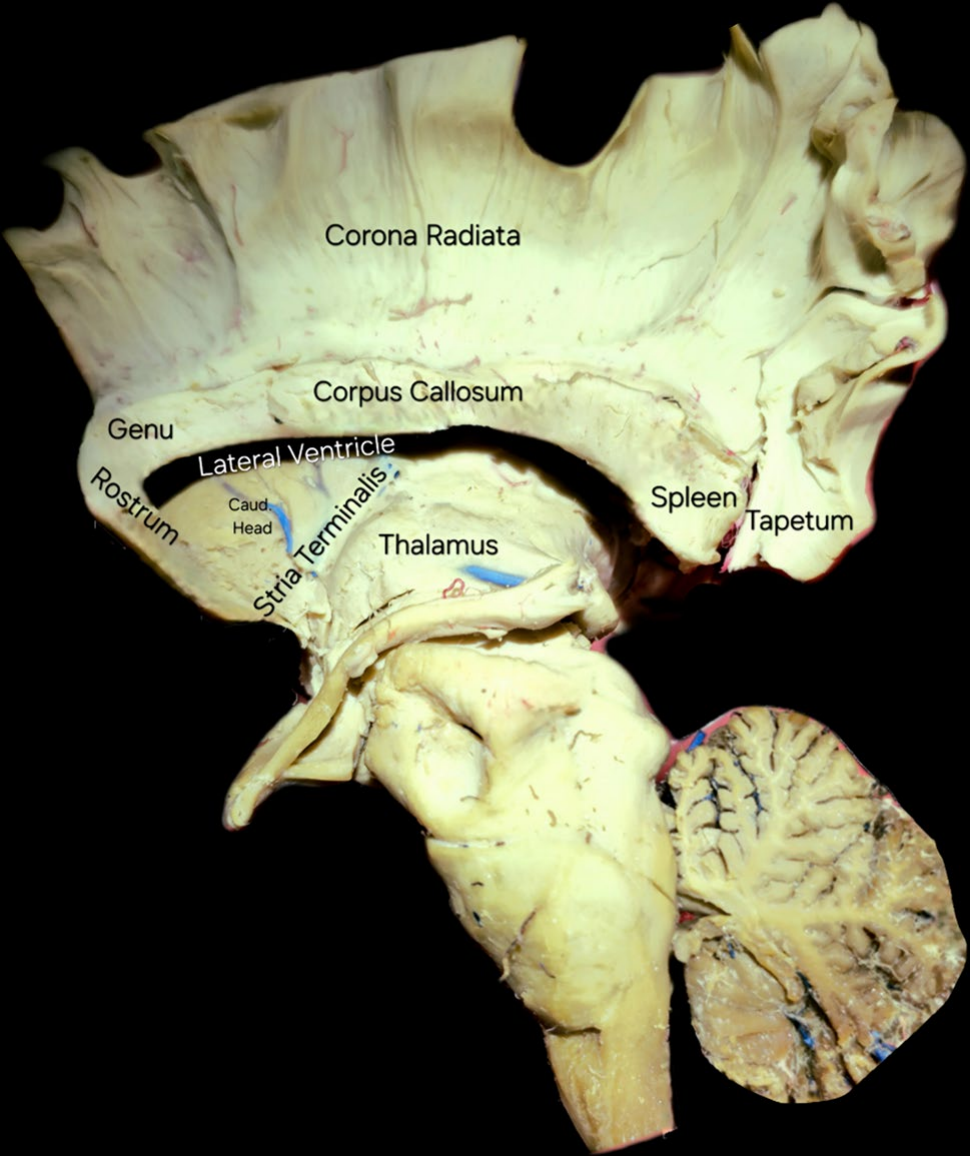
Deep structures of the Central Core, Corpus Callosum and Brainstem from a lateral view, many of which are supplied anteriorly by perforators arising from A1, ACommA and M1, through the anterior perforated substance. White matter dissection was conducted in order to demonstrate the projection of nervous fibers

Regarding the perforating branches, the MCA provides an average of 10 per hemisphere, divided into medial lenticulostriate, intermediate lenticulostriate and lateral lenticulostriate. These are responsible for irrigation of part of the internal capsule and substantial portion of the basal ganglia [1] (Fig. 10).

## Supratentorial venous system

The encephalic venous system is characteristically variable in nature—caliber and connection-wise. One must discriminate between the supratentorial venous system and the posterior fossa venous system, in order to accommodate all the data and evidence that exist in a logical, intuitive scheme [26]. The veins of the supratentorial space, can be classified in two groups: superficial and deep. The veins of the posterior fossa are divided into four groups: superficial, deep, brainstem, and bridging veins. The Internal Jugular Veins (IJVs) are the major source of outflow of blood from the intracranial compartment, the right one being commonly dominant [48, 49]. Other lesser sources of outflow include orbital veins and the venous plexuses around the vertebral arteries. Sometimes, even diploic and scalp veins act as collateral pathways [14]. Nevertheless, for its conspicuousness and great capacitance caliber, the satisfactory functioning of the IJV is paramount for encephalic homeostasis, although not the only outflow pathway, such as the conspicuous but often forgotten pterygopalatine venous plexus [32, 33, 45].

Superficial groups drain the cortical surface, whereas deep groups drain the neural structures in the vicinity of the ventricles and the basal cisterns. The superficial group collects into four subgroups of bridging veins: a superior sagittal group that drains into the superior sagittal sinus; a sphenoidal group that drains into the sphenoparietal or cavernous sinus; a tentorial group that converges on the sinuses in the tentorium; and a falcine group that drains into the inferior sagittal or straight sinus. The deep system drains into veins that course near the ventricular system and basal cisterns and converge on the internal cerebral, basal, and great veins and their tributaries. They are divided into a ventricular group, comprised of veins draining the walls of the lateral ventricles, and a cisternal group, which includes the veins draining the walls of the basal cisterns. The internal cerebral vein is included in the ventricular group, because it is predominantly related to the ventricles. The basal and great veins, as well as the choroidal veins, are also included with the ventricular veins. The thalamic veins, however, are included in both the ventricular and the cisternal groups, because they run across the ventricular surface and others course through the basal cisterns [20, 42, 46].

Last but not least, one must also be aware of the existence of the Diploic Venous System, which is located between the two hard compact layers of cranial bone and immersed in its cancellous bone, called *diploë*. It has been demonstrated that several patterns of distribution and localization of the DVS exist in a systematic fashion, classifying the DVS into 7 types: bonsai, spider, coronal, serpentine, thousand lakes, mixed, and undetermined. Later on, García-González, U et al. demonstrated and majorly accurate location of the main diploic veins. Thus, revealing that the draining point of the frontal diploic vein (FDV) was located near the supraorbital notch; the draining point of the anterior temporal diploic vein (ATDV) was located in all pterional areas; and the draining point of the posterior temporal diploic vein (PTDV) was located in all asterional areas. The latter, being the dominant diploic vessel in all sides [11, 14].

## Limitations and strengths

The present work provides a succinct yet comprehensive review of the fundamentals that every novel scholar in advanced neuroanatomy must know. For the sake of brevity, the following systems have not been included in this paper: Vertebral plexuses, External Carotid or External Jugular Vein. Advanced vascular morphometry has also not been discussed. Nevertheless, this paper provides a straightforward approach to this complex subject matter. We humbly believe that, although there are cornerstone studies that delve into neurovascular anatomy, our work tackles the matter in a way that, while compelling and thorough, does not overwhelm the novel reader.

## Conclusion

**Fig. 11:**
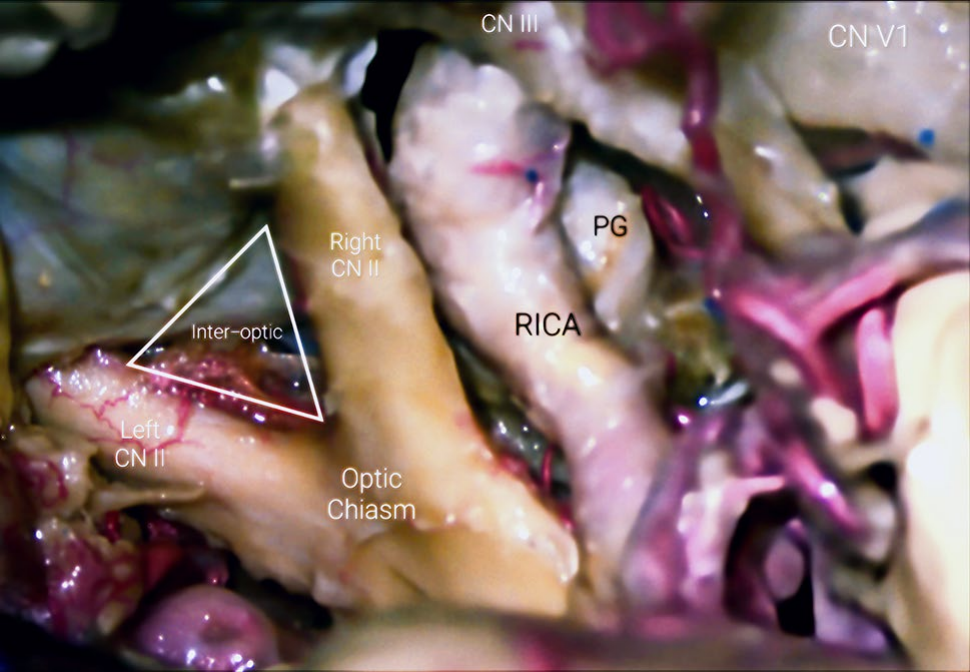
Surgical view of main neurovascular structures through a right Fronto-Temporal approach

There already is fundamental literature concerning this topic, on which this endeavor is based upon. This work serves as a preliminary but thorough guide, providing essential knowledge on the neurovascular anatomy of the encephalon. It aims to aid in the understanding of the intricate vasculature of the brain, which is crucial for neurosurgical and neurology related practice regarding clinical decision-making. The purpose of these lines above is to provide an early framework for vascular neuroanatomy, thus enhancing whatever initial knowledge novice physicians might have on that subject, henceforth making more efficient the use of available time. It is meant to be a work to be kept in the armamentarium of training scholars and neuroanatomists. Figure 11**.**

## Data Availability

The authors confirm that the data supporting the findings of this study are available within the article.
